# Cross-Fostering of Male Mice Subtly Affects Female Olfactory Preferences

**DOI:** 10.1371/journal.pone.0146662

**Published:** 2016-01-12

**Authors:** Ying-Juan Liu, Yao-Hua Zhang, Lai-Fu Li, Rui-Qing Du, Jin-Hua Zhang, Jian-Xu Zhang

**Affiliations:** 1 School of Life Science and Technology, Nanyang Normal University, Nanyang, Henan Province, China; 2 State Key Laboratory of Integrated Management of Pest Insects and Rodents in Agriculture, Institute of Zoology, Chinese Academy of Sciences, Beijing, China; CNRS, FRANCE

## Abstract

The maternal environment has been shown to influence female olfactory preferences through early chemosensory experience. However, little is known about the influence of the maternal environment on chemosignals. In this study, we used two inbred mouse strains, C57BL/6 (C57) and BALB/c (BALB), and explored whether adoption could alter male chemosignals and thus influence female olfactory preferences. In Experiment 1, C57 pups were placed with BALB dams. Adult BALB females then served as the subjects in binary choice tests between paired male urine odours (BALB vs. C57, BALB vs. adopted C57 and C57 vs. adopted C57). In Experiment 2, BALB pups were placed with C57 dams, and C57 females served as the subjects in binary choice tests between paired male urine odours (C57 vs. BALB, C57 vs. adopted BALB, and BALB vs. adopted BALB). In both experiments, we found that females preferred the urine of males from different genetic backgrounds, suggesting that female olfactory preferences may be driven by genetic compatibility. Cross-fostering had subtle effects on female olfactory preferences. Although the females showed no preference between the urine odours of adopted and non-adopted males of the other strain, the BALB females preferred the urine odour of BALB males to that of adopted C57 males, whereas the C57 females showed no preference between the urine odour of C57 and adopted BALB males. Using gas chromatography-mass spectrometry (GC-MS) and stepwise discriminant analysis, we found that the ratios of volatile chemicals from urine and preputial gland secretions were altered in the fostered male mice; these changes may have resulted in the behavioural changes observed in the females. Overall, the results suggest that female mice prefer urine odours from males with different genetic backgrounds; this preference may be driven by genetic compatibility. The early maternal environment influences the chemosignals of males and thus may influence the olfactory preferences of females. Our study provides additional evidence in support of genotype-dependent maternal influences on phenotypic variability in adulthood.

## Introduction

In nature, alloparental care and adoption have been documented in hundreds of mammalian and avian species [1]. Maternal provisioning can influence a wide range of traits including development, behaviour, fitness, and epigenetic changes [2–7]. Maternal effects can sometimes result in phenotypic similarities between foster mothers and offspring, including similarities in microbial communities, vocalizations, and the expression of emotion [8–11]. However, maternal effects are plastic, and maternal provisioning is often influenced by the genotype of the offspring [2–7,12]. Moreover, males are more susceptible to maternal effects than females [8,13].

In some rodent species, olfactory-mediated kin recognition is affected by the maternal environment, such that genotypic cues are learned through early chemosensory experiences. For example, females avoid mating with males carrying the major histocompatibility complex (MHC) alleles of their foster family rather than of their natural family, suggesting that familial imprinting determines their preferences [2,14]. In rodents, female olfactory preferences primarily rely on the assessment of male chemosignals, but whether the maternal environment can alter the chemosignals of males and consequently influence female choice remains unknown.

The mouse is the most studied mammalian species with regard to chemosignals. Several volatile male pheromones have been identified from bladder urine and the preputial glands that have clear biological effects, such as attracting females [15]. Urinary volatiles are derived from bladder urine and preputial gland secretions (PGSs). These compounds not only co-vary with genetic distance but also are affected by social conditions and convey information about kinship, social and physical status to potential mates; thus, they may influence mate choice decisions by female mice [15,16].

The individuals of an inbred strain are almost identical in both genotype and phenotype and thus provide standardized animal models for studies of kin recognition and mate choice [14,17,18]. Two inbred mouse strains, C57BL/6 (C57) and BALB/c (BALB), have been widely used in studies of gene-environment interactions and genetic-related chemosignals. These strains are genetically distinct and differ with regard to maternal care, reactivity to stress, and anxious behaviour [5,18–22]. The chemosignals derived from the urine and PGSs of the two strains are also quantitatively different and thus facilitate the avoidance of inbreeding [16–18].

Behavioural tests and chemical analyses are indispensable tools for deciphering complex chemosignals [23]. In this study, by combining binary choice tests and gas chromatography-mass spectrometry (GC-MS), we examined whether the maternal environment can influence male chemosignals and thus influence female olfactory preferences. We investigated whether BALB female olfactory preferences between BALB male urine and control C57 male urine were the same as those between the urine of BALB males and adopted C57 males, or vice versa (whether C57 female olfactory preferences between C57 male urine and BALB male urine were the same as those between the urine of C57 males and adopted BALB males). The urine-borne volatiles, which contain both metabolized urine volatiles and PGS volatiles, were also analysed.

## Materials and Methods

### Animals

BALB and C57 mice (Vital River Laboratories, Beijing, China) were housed in male-female pairs of the same strain for breeding (cage dimensions 25 × 15 × 13.5 cm). Males were removed once the females were noticeably pregnant. All mice were raised in environments with a reversed 14 light:10 dark photoperiod at 22 ± 2°C and were provided with food and water ad libitum. All experiments were carried out during the dark period from 9:00 to 19:00 under dim red light.

### Ethical standards

The procedures for animal care and use in this study were fully compliant with the legal requirements of China and were approved by the Animal Use Committee of the Institute of Zoology, Chinese Academy of Sciences (approval number IOZ12068), where the experiments were conducted. All efforts were made to minimize suffering during the experiments.

### Adoption procedure

The adoption procedures were performed 4–7 h after parturition. In experiment 1, entire C57 litters were assigned to BALB mothers. Specifically, after the biological mother was removed, the C57 pups were counted, sexed, thoroughly mixed with the BALB mother’s bedding and then placed in a clean cage with the BALB mother. The entire procedure required less than 20 min. Control C57 pups were treated in the same manner but were caged with their biological mother. The pups were weaned at 28 days of age, housed in same-sex sibling groups (no more than 4 mice per cage) and left undisturbed until 12 weeks of age for further study [2,5,6,19]. In experiment 2, the approach was nearly the same, except that BALB litters were cross-fostered to C57 mothers.

Five C57 litters (6–9 pups per litter) were raised by BALB dams in experiment 1, five BALB litters were raised by C57 dams in experiment 2, and 8 BALB and 7 C57 litters were raised by their biological mothers. Thirteen adopted C57 males, twelve adopted BALB males, and 21 control C57 and BALB males served as odour donors. BALB females (experiment 1) and C57 (experiment 2) females in oestrus served as subjects (odour recipients).

### Urine collection

Each donor mouse was placed in a clean mouse cage (25 × 15 × 13.5 cm) with a wire grid floor 1 cm above the bottom. After the mice had urinated, the urine was immediately collected using a disposable glass capillary tube (i.d. 1.8 mm, 15 cm long) and transferred to a vial held on ice. Urine was collected from each mouse once a day and individually sealed and stored at −20°C until further use.

### PGSs preparations

After the urine was collected, the mice were transferred to a room adjacent to the laboratory, where they were euthanized via the injection of sodium pentobarbital (100 mg/kg, i.p.). The paired preputial glands were immediately excised and weighed; PGSs were then collected in a clear vial by squeezing the glands. The PGS samples were stored individually and held at −20°C for further chemical analysis.

### Chemical assays

Samples were extracted before chemical analysis using previously described methods [16]. Prior to extraction, each urine sample and PGS sample was thawed at room temperature. An Agilent Technologies Network 6890 GC system coupled with a 5973 Mass Selective Detector (NIST 2002 Library) was used for qualitative analysis, and an Agilent 7890N gas chromatograph equipped with a flame ionization detector was used for quantitative analysis [15,16,24]. The gas chromatograph was equipped with an HP5 MS glass capillary column (30 m × 0.25 mm i.d., film thickness 0.30 μm). The inlet temperature was 230°C. The oven temperature was initially set at 50°C (for urine) or 100°C (for PGSs), and then, the oven was heated at a rate of 5°C /min to 150°C (for urine) or 230°C (for PGSs). Four microliters of urine or 2 μl PGS extract was injected in a splitless mode.

**Fig 1:**
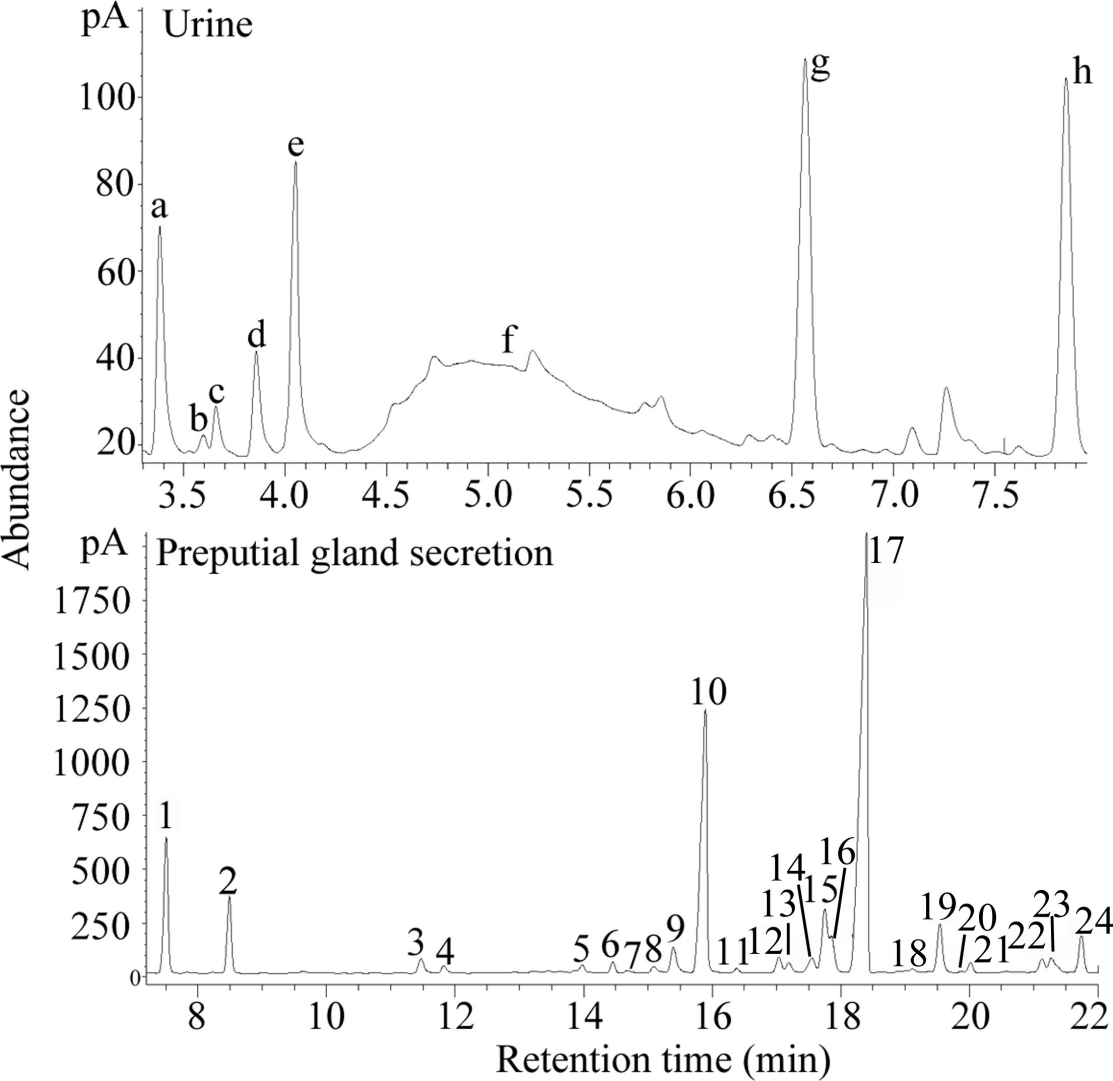
Representative gas chromatogram of volatile compounds from urine and preputial gland secretions. Compound numbers and letters correspond to those used in S1 Table.

We characterized the compounds by comparing the retention times with those of standard compounds (J&K Chemical Ltd, Beijing, China) and by matching the mass spectra with those in the NIST 2002 Library [16,25]. The relative abundance of each compound (each GC peak area/summed GC peak area × 100) was used in the statistical analysis (Fig 1).

### Binary choice test

We injected a 2 μl urine sample into a disposable glass capillary tube (i.d. 1.1–1.2 mm, o.d. 1.3–1.4 mm, 15 cm length), which was then sealed with odourless gum at one end. The sample remained inside the capillary tube, 1 cm from the capillary tip, so that the mice could not come into direct contact with the sample [26].

Vaginal smears were taken from all females (BALB in experiment 1 and C57 in experiment 2) to determine the female’s stage in the oestrous cycle. Only female mice in oestrus were used in the following experiments. The oestrus state was determined according to the procedures of Byers at least 2 h before testing [27]. The female was left in the home cage for the test, while its cage mate was temporarily moved into an identical holding cage (the olfactory preference tests were conducted in the home cage to minimize the effects of manipulation and environmental stimuli on behaviour). We presented the female with paired urine samples (BALB vs. C57, BALB vs. adopted C57 and C57 vs. adopted C57 in experiment 1; C57 vs. BALB, C57 vs. adopted BALB, and BALB vs. adopted BALB in experiment 2) and recorded the total time the female spent investigating the samples over a 3 min period that was begun after the subject first sniffed or licked the capillary tip. Each subject was used only once per day, and total times of less than one second were excluded from analysis.

### Statistical analysis

We analysed the behavioural data using either paired t test or Wilcoxon matched-pairs signed-rank test (for non-normal data). The level of significance was set at 0.05 (SPSS Version 15.0).

To identify the chemical compositions associated with the behavioural changes, we used discriminant analysis techniques (stepwise discriminant analysis) to analyse the GC-MS data; this type of analysis is commonly used to differentiate three groups of samples [28]. The analysis is described in detail below.

In experiment 1, we first analysed differences in the chemical composition of the urine and PGSs between the control C57 and adopted C57 groups. If the chemical compositions were more than 95% similar, we assumed that there was no significant difference between the two groups and merged them into a new group, which was designated as the combined C57 (cC57) group.

For the first level of screening, we identified which components differed between the BALB and cC57 groups and designated these as *A*_*1*_, *A*_*2*_, *A*_*3*,_ …, *A*_*n*._ We then determined whether the two groups could be fully differentiated based on the selected components. If the recognition rate was 100%, the selected components were used for the next step in the analysis. If the recognition rate was less than 100%, a second round of selection from *A*_*1*_, *A*_*2*_, *A*_*3*_, … *A*_*n*,_ was conducted to identify appropriate components, which were then designated *B*_*1*_, *B*_*2*_, … *B*_*m*_ (with a recognition rate of 100%).

For the second level of screening, we determined which of the selected components (*A*_*1*_, *A*_*2*_, *A*_*3*_, … *A*_*n*_ or *B*_*1*_, *B*_*2*_, … *B*_*m*_) fully differentiated BALB vs. C57 and BALB vs. adopted C57 and designated them as *C*_*1*_, *C*_*2*_, *C*_*3*_, … *C*_*t*_ and *D*_*1*_, *D*_*2*_, *D*_*3*_, … *D*_*k*_, respectively.

For the third level of screening, the components that were common between *C*_*1*_, *C*_*2*_, *C*_*3*_, … *C*_*t*_ and *D*_*1*_, *D*_*2*_, *D*_*3*_, … *D*_*k*_ were designated as *E*_*1*_, *E*_*2*_, *E*_*3*_, … *E*_*f*,_, and we determined whether *E*_*1*_, *E*_*2*_, *E*_*3*_, … *E*_*f*_ could fully differentiate all three of the comparison groups, i.e., BALB vs. cC57, BALB vs. C57 and BALB vs. adopted C57. If all of the recognition rates were 100%, we assumed that *E*_*1*_, *E*_*2*_, *E*_*3*_, … *E*_*f*_ were characteristic components that fully discriminated the BALB, C57 and adopted C57 groups. If the recognition rate was not 100%, then we repeated the 2^nd^ and 3^rd^ level screenings based on *E*_*1*_, *E*_*2*_, *E*_*3*_, … *E*_*f*_ to determine the final characteristic components (for which recognition rates were 100%).

Using the same method, we selected the characteristic components that fully discriminated among C57, BALB and adopted BALB groups in experiment 2.

## Results

### Olfactory preferences of females

**Fig 2:**
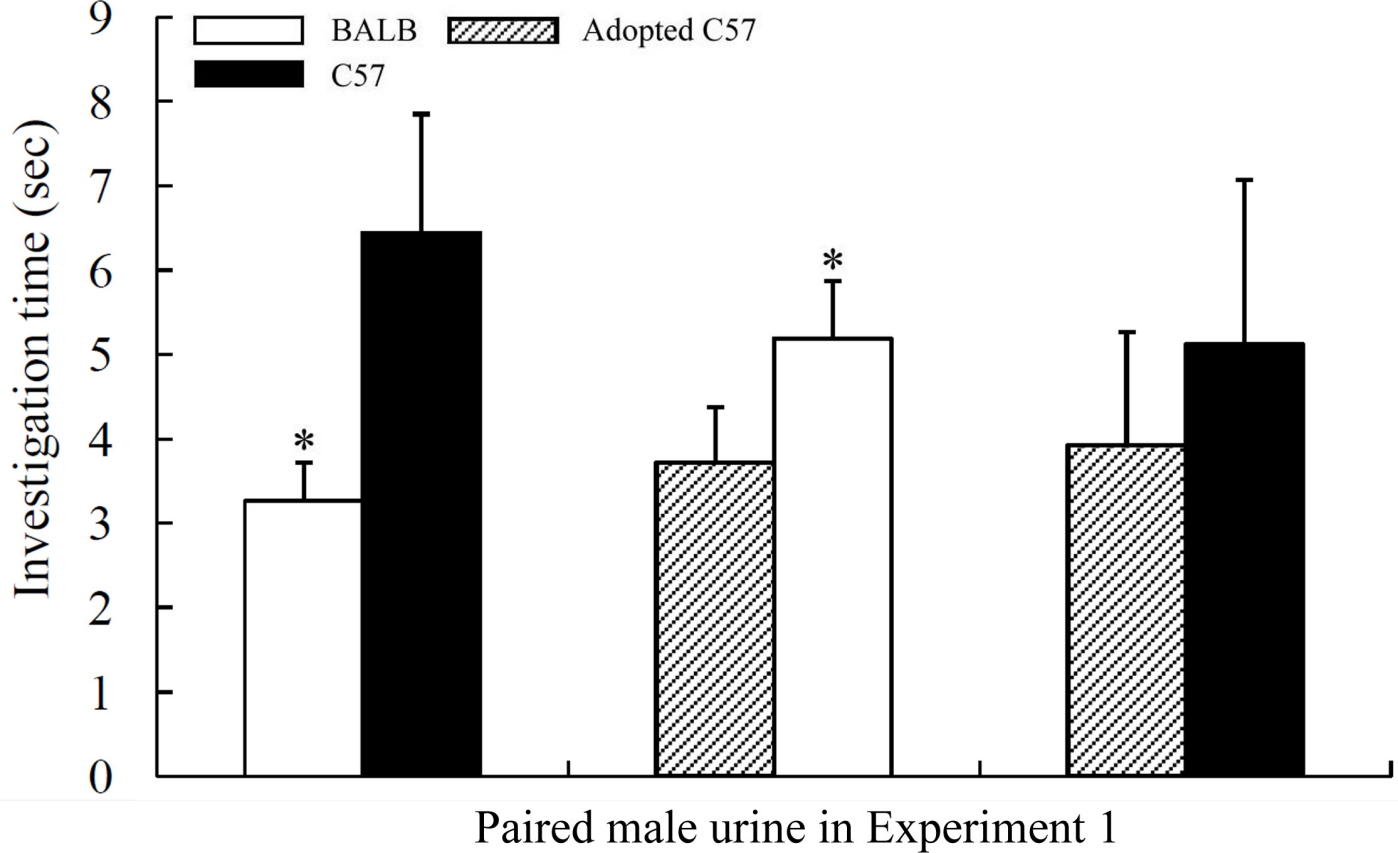
Investigation times of BALB females to different male urine samples during the 3 min binary choice tests. Mean ± SE, n = 11 or 12, *P < 0.05, Wilcoxon signed-rank test or paired t test.

**Fig 3:**
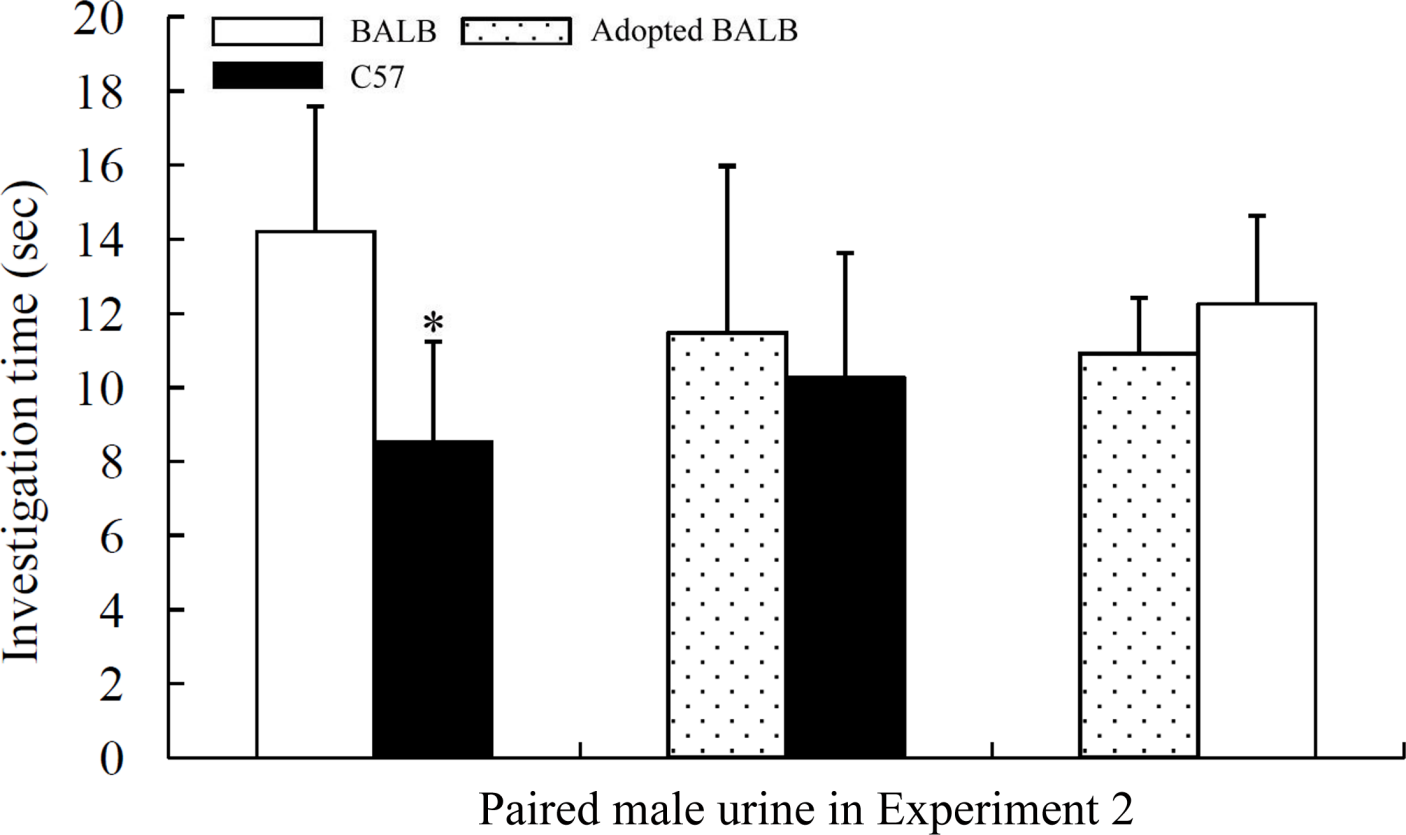
Investigation times of C57 females to different male urine samples during the 3 min binary choice tests. Mean ± SE, n = 10–15, *P < 0.05, Wilcoxon signed-rank test or paired t test.

Binary choice tests revealed that the females preferred the urine from males of a different genetic background. In Experiment 1, the BALB female mice were more attracted to control C57 male urine than to BALB male urine (neither group was cross-fostered, left bar in Fig 2; paired t test: t = 2.373, P = 0.039, n = 11); in Experiment 2, the C57 females were more attracted to BALB male urine (neither group was cross-fostered, left bar in Fig 3; Z = 1.789, P = 0.047, n = 14). These results suggest that female olfactory preferences may be driven by genetic compatibility.

Cross-fostering subtly affected female olfactory preferences. Although the females showed no preference between the urine odours of adopted and non-adopted males of the same strain (Experiment 1: C57 vs. adopted C57, Wilcoxon signed-rank test: Z = 0.392, P = 0.695, n = 12, right bar in Fig 2; Experiment 2: BALB vs. adopted BALB, Paired t test: t = 0.752, P = 0.471, n = 10, right bar in Fig 3), the BALB females preferred the urine odour of BALB males to that of adopted C57 males in Experiment 1 (Paired t test: t = 2.371, P = 0.037, n = 12, middle bar in Fig 2), but the C57 females showed no preference between the urine odour of the C57 and adopted BALB males in Experiment 2 (Wilcoxon signed-rank test: Z = 0.398, P = 0.691, n = 15, middle bar in Fig 3).

### Volatile profiles of male urine and PGSs

The relative abundances of each compound identified in the urine and PGSs are listed in S1 Table. We then used stepwise discriminant analysis to identify the components involved in the process of female choice. The entire analytical process is presented in S1 File.

#### Characteristic components in Experiment 1

Using the aforementioned analytical method, the selected components were 5, 16, 18, 19, and 23 from PGSs and c and g from urine (the numbers and letters correspond to the chromatograph peaks, as shown in Fig 1). All of these components could fully differentiate the three comparison groups, i.e., BALB vs. cC57, BALB vs. C57 and BALB vs. adopted C57 (recognition rates were 100%). The relative abundance of each of the seven selected components is listed in Table 1.

**Table 1:** Relative abundances of the characteristic components selected in Experiment 1 (mean ± SD).*

Groups	PGSs					Urine	
	5	16	18	19	23	c	g
**BALB**	0.51±0.12	2.40 ± 0.20	0.25 ± 0.02	3.43 ± 0.40	1.39 ± 0.27	0.37 ± 0.17	8.02 ± 4.20
**C57**	0.81 ± 0.22	2.13 ± 0.29	0.11 ± 0.02	1.97 ± 0.37	0.95 ± 0.39	1.23 ± 0.93	3.92 ± 1.91
**Adopted C57**	0.66 ± 0.23	2.16 ± 0.11	0.12 ± 0.02	2.18 ± 0.17	1.17 ± 0.26	1.07 ± 0.76	2.58 ± 1.07

* Compound numbers and letters correspond to those used in S1 Table and Fig 1. PGSs: preputial gland secretions.

#### Characteristic components in Experiment 2

In Experiment 2, the selected characteristic components were identified as 3, 5, 18, 19 and 23 from PGS and a, c and g from urine (the numbers and letters correspond to the chromatograph peaks, as shown in Fig 1). All of these components could fully differentiate the three comparison groups, i.e., C57 vs. cBALB, BALB vs. C57 and BALB vs. adopted BALB (recognition rates of 100%). The relative abundance of each of the eight characteristic components is listed in Table 2.

**Table 2:** Relative abundances of the characteristic components selected in Experiment 2 (mean ± SD).*

Groups	PGSs					Urine		
	3	5	18	19	23	a	c	g
**C57**	2.05 ± 1.11	0.81 ± 0.22	0.11 ± 0.02	1.97 ± 0.37	0.95 ± 0.39	4.07 ± 0.85	1.23 ± 0.93	3.92 ± 1.91
**BALB**	1.00 ± 0.51	0.51 ± 0.12	0.25 ± 0.02	3.43 ± 0.40	1.39 ± 0.27	6.66 ± 2.25	0.37 ± 0.17	8.02 ± 4.20
**Adopted BALB**	1.08 ± 0.61	0.45 ± 0.14	0.27 ± 0.04	3.29 ± 0.37	1.59 ± 0.38	7.43 ± 2.32	0.49 ± 0.26	8.33 ± 4.24

* Compound numbers and letters correspond to those used in S1 Table and Fig 1. PGSs: preputial gland secretions.

Six characteristic components were shared between Experiments 1 and 2 (Tables 1 and 2): 5, 18, 19 and 23 from PGSs and c and g from urine (the corresponding chemical compounds were Z-5-tetradecenol-1-ol, 1-heptadecanol acetate, 1-heptadecanol acetate [branched], Z-7-octadecen-1-ol acetate, 2-heptanone, and R,R-3,4-dehydro-exo-brevicomin [DHB], respectively). The identification of common characteristic components may indicate that our selection process was reasonable and effective. Alternatively, it might indicate that the six characteristic components played an important role in the difference in investigation times among the paired odours.

## Discussion

In this study, we used two inbred strains of mice (C57 and BALB) and found that females preferred the urine of males from a different genetic background, suggesting that female olfactory preferences may be driven by genetic compatibility. However, the BALB females preferred the urine of BALB males over adopted C57 males, whereas the C57 females showed no preference between C57 and adopted BALB male urine. Using GC-MS in addition to stepwise discriminant analysis, we found that the composition of chemosignals derived from urine and PGSs was altered by cross-fostering, and the compositional alterations may have played an important role in the differences in investigation time among the paired odours.

### Genotype-odour type correlations and preferences for genetic compatibility

In mammals, the chemosignals that serve as kinship cues often consist of a large number of compounds in gestalt or mosaic forms [29,30]. In this study, we found significant differences in many of the compounds derived from urine and PGSs between the two tested strains, consistent with previous reports that the two strains have different genetic backgrounds [16–18]. The females preferred the urine from males of a different genetic background; this preference may have been driven by genetic compatibility or inbreeding avoidance [2,14,31]. Thus, urinary volatiles may provide information about an individual’s genetic identity that could be used during mate selection [32,33], just as major urinary proteins (MUPs) and MHCs have been shown to do [34,35].

However, our results were not consistent with the study of Yano [36], which showed that the preference of BALB/c females for the urine of C57 males over BALB/c males changed with their oestrus cycle; the urine of C57 males was only preferred when the females were in a non-oestrus state. The reasons for this inconsistency remain unknown, although we suspect that differences in the test environment, urine samples, and the duration of the test (3 min in our study compared with 10 min in Yano’s study) may have contributed to the discrepancy. More strictly controlled studies should be conducted to further evaluate the relation between female olfactory preferences and the oestrus cycle.

### Cross-fostering and female olfactory preferences

Our results indicate that strain cues were not disrupted by the altered maternal environment [2,14,24,33,37]. Accordingly, the BALB females responded similarly to the urine of control C57 and adopted C57 males, and the C57 females responded similarly to the urine of control and adopted BALB males. Therefore, genetic influences on odour type appear to be stable and persistent, as reported in mice and non-cohabiting human twins [38].

However, our results showed that the change in maternal environment did subtly affect the mate-attraction process. In choices between the urine of BALB and adopted C57 males, the BALB females preferred BALB males, which have a similar genetic background, but the C57 females showed no preference between the urine of C57 males and adopted BALB males. Cross-fostering differentially affected the two strains. It is intuitive to think that gene × environment interactions may account for this discrepancy. Previous studies have shown that the two strains differ with respect to levels of maternal care, stress reactivity, and anxiety-like behaviour in adulthood [19,20]. Priebe et al. reported that cross-fostering differentially affected anxiety-like behaviour and basal corticosterone levels in the two strains [5]. However, determining which factors, e.g., maternal care, emotional or hormonal changes, are responsible for the discrepancy requires further investigation.

The altered olfactory preferences also appear to contradict the “inbreeding avoidance” rules described above. Thus, the odours of individual male mice seem to be determined by both genetic and early environmental factors. This finding contrasts with male songs, which are determined only by genetic factors [21]. However, we cannot exclude the possibility that other factors contributed to this process because female olfactory preference in mice is a complex process; inbreeding avoidance contributes only when there is little variation in the genetic quality of potential mates [37].

As noted, the early maternal environment is known to critically influence neural, hormonal, and behavioural outcomes in rodents [5,39]. Some sexually attractive chemicals in the urine and PGSs are normally under the control of sex hormones [15,40]. Thus, it is possible that in the current study, the altered early maternal environment influenced the hormonal status of the animals as adults, changing the composition ratio of some sexually attractive chemicals in the urine and ultimately altering the olfactory preference of the female mice. Consistent with this idea, Bartolomucci et al. reported that the weight of the preputial glands was reduced in cross-fostered male mice; these glands are the main source of pheromones [8].

Using GC-MS and stepwise discriminant analysis, we identified six chemosignals that may have played an important role in the differences in investigation time among the paired odours. Of these chemicals, Z-5-tetradecenol-1-ol acetate and DHB have been shown to be sexually attractive to females, while 2-heptanone is associated with oestrous in females [15,34,41]. The other three chemicals have not been reported to be related to sexual attraction and require further examination in future chemical and behavioural studies. While we do not think one particular volatile plays an essential role in this process, the selected airborne volatiles may function as a whole in chemical communication. Therefore, we propose the following mechanism for the attractiveness variations induced by cross-fostering: in Experiment 1, the change in maternal environment subtly attenuated the relative abundance of 2-heptanone and DHB and, at the same time, elevated the relative abundance of 1-heptadecanol acetate (branched) and Z-7-octadecen-1-ol acetate; these changes may have contributed to the preference of the BALB females for BALB rather than adopted C57 urine (the correct choice based on genetic differences). Similarly, the elevated relative abundance of Z-7-octadecen-1-ol acetate, 2-heptanone, and DHB and the attenuated abundance of 1-heptadecanol acetate (branched) and Z-5-tetradecenol acetate in the urine of the adopted BALB males may have resulted in the C57 females showing no preference between C57 and adopted BALB males in Experiment 2. However, we cannot exclude the possibility that other chemical signals are involved in this process.

MUPs are known to be necessary and sufficient for kin recognition and mate choice in mice [42,43]. The MUP darcin (18893Da) has been shown to promote innate sexual attraction in female mice [44,45]. The lack of measurements of changes in MUPs is a limitation of the current study. Regardless, the effects of proteins are limited to direct contact; olfactory assessment of volatile chemosignals may serve as the first step in chemical communication during the process of mate selection. Although some studies have suggested that male airborne volatiles became attractive to female mice only when repeatedly associated with MUPs [46,47], increasing evidence indicates that attraction to urinary odours in rodents likely involves the recognition of both airborne and non-volatile chemicals, which requires the integration of both the olfactory and vomeronasal systems (reviewed by Fortes-Marco, et al. [48]).

Another limitation of this study is that we did not analyse the impact of adopting within the same strain (intrastrain cross-fostering); this shortcoming prevented us from interpreting whether the behavioural differences were due to strain, cross-fostering or some interaction between the two. However, inbred mouse strains are thought to be genetically identical, and studies have shown that intrastrain cross-fostering minimally affects maternal provisioning [6,39]. Thus, it is reasonable to speculate that the variations are due to cross-fostering. If so, the olfactory preferences of females to intrastrain cross-fostered males should be the same as those to non-cross-fostered control males.

### Conclusions

In conclusion, we showed for the first time that cross-fostering alters the composition of some airborne chemicals, which could lead to changes in the olfactory preferences of females. Our study provides new understanding of how changes in the maternal environment influence the olfactory preferences of female mice (an important step in mate choice), which is another piece of evidence in support of genotype-dependent maternal influences on phenotypic variability in adulthood. Future studies should examine whether the six selected chemosignals are actually involved in mate choice in mice or whether other chemicals, e.g., non-volatile MUPs, are involved in chemically mediated mate selection. It would also be interesting to investigate whether there are gender differences in this process.

## Supporting Information

S1 File The process of the progressive discriminant analysis. (DOC)

S2 File Language editing certificate of American Journal Experts. (PDF)

S1 Table Relative abundance of preputial gland secretions (PGSs) and urinary volatiles in the four groups of male mice. (DOC)
