# Supplementary material for: Cross-Fostering of Male Mice Subtly Affects Female Olfactory Preferences
Source: PLoS One. 2016 Jan 12;11(1):e0146662. doi: 10.1371/journal.pone.0146662 (PMC4710493; doi:10.1371/journal.pone.0146662)
Supplement: S1 File — (DOC) [file pone.0146662.s001.doc]

**Cascade discriminant analysis**

***The selection process in Experiment 1***

Using discriminate analysis, we found there was no significant difference of the 32 chemicals [8 from urine, 24 from preputial glands secretions (PGSs)] between C57 and adopted C57 groups. In odor to maximize the variations, we merged them into a new group, which was designated as the combined C57 (cC57) group. The results were list in Table A1.

For the first level of screening, we identified which components differed between the BALB and cC57 groups using Wilks' Lambda test. We found components 3, 5, 7, 8, 16, 18, 19, 22, 23, a, c and g were significantly different between the two groups (Table A2); all the 12 components could fully differentiate the two groups (recognition rates were 100%, Table A3).

For the second level screening, base on the above selected 12 components, we found components 3, 5, 16, 18, 19, 23, a, c and g were significant different between BALB and C75 groups (7, 8, 22 were not significant), (Table A4); components 5, 7, 8, 16, 18, 19, 22, 23, c and g were significant different between BALB and adopted C75 groups (3 and a were not significant), (Table A5). The shared components were 5, 16, 18, 19, 23, c and g.

For the third level screening, based on the selected shared seven components, we found all of them could fully differentiate all the three comparison groups, i.e., BALB vs. cC57, BALB vs. C57 and BALB vs. adopted C57 (recognition rates were 100%), (Table A6-A8). The relative abundances of these components were listed in Table 1.

***The selection process in Experiment 2***

Using discriminate analysis, we found there was only one ingredient was different between the BALB and adopted BALB group (Table A9). The chemical compositions were more than 95% similar. Similarly, in odor to maximize the variations, we merged the two groups into a combined group designated as cBALB group.

For the first level screening, we found components 3, 4, 5, 8, 12, 14, 15, 16, 18, 19, 21, 22, 23, a, b, c, d and g were significant different between C5 and cBALB groups (Table A10). All the 18 components could fully differentiate the two groups (recognition rates were 100%), (Table A11).

For the second level screening, base on the selected 18 components, we found components 3, 5, 16, 18, 19, 23, a, c, and g were significant different between the BALB and C75 groups (8, 12, 14, 15, 21, 22, b and d were not significant), (Table A12); components 3, 5, 8, 18, 19, 22, 23, a, c, d, and g were significant different between C57 and adopted BALB (4, 12, 14, 15, 16, 21 and b were not significant), (Table A13). The shared components were 3, 5, 18, 19, 23, a, c and g.

For the third level screening, based on the selected 8 components, we found all of them could fully differentiate all the three comparison groups, i.e., C57 vs. cBALB, C57 vs. BALB and C57 vs. adopted BALB (recognition rates were 100%), (Table A14-A16). Then, components 3, 5, 18, 19, 23 from PGS, and a, c and g from urine were characteristic components in experiment 2. The relative abundances of these components were listed in Table 2.

**Table A1. Tests of equality of group means (C57 vs. adopted C57).**

|  | **Wilks' Lambda** | **F** | **df1** | **df2** | **Sig.** |
| --- | --- | --- | --- | --- | --- |
| **Chemical components from PGS** |  |  |  |  |  |
| 1 | 1 | 0 | 1 | 13 | 0.996 |
| 2 | 1 | 0 | 1 | 13 | 0.986 |
| 3 | 0.969 | 0.41 | 1 | 13 | 0.533 |
| 4 | 0.964 | 0.491 | 1 | 13 | 0.496 |
| 5 | 0.977 | 0.3 | 1 | 13 | 0.593 |
| 6 | 1 | 0.004 | 1 | 13 | 0.949 |
| 7 | 0.968 | 0.426 | 1 | 13 | 0.525 |
| 8 | 0.991 | 0.123 | 1 | 13 | 0.732 |
| 9 | 1 | 0.006 | 1 | 13 | 0.941 |
| 10 | 1 | 0 | 1 | 13 | 0.997 |
| 11 | 0.89 | 1.604 | 1 | 13 | 0.228 |
| 12 | 0.917 | 1.174 | 1 | 13 | 0.298 |
| 13 | 0.921 | 1.11 | 1 | 13 | 0.311 |
| 14 | 0.821 | 2.835 | 1 | 13 | 0.116 |
| 15 | 0.996 | 0.054 | 1 | 13 | 0.819 |
| 16 | 0.999 | 0.015 | 1 | 13 | 0.905 |
| 17 | 0.941 | 0.812 | 1 | 13 | 0.384 |
| 18 | 0.85 | 2.301 | 1 | 13 | 0.153 |
| 19 | 0.911 | 1.263 | 1 | 13 | 0.281 |
| 20 | 1 | 0 | 1 | 13 | 0.998 |
| 21 | 0.861 | 2.104 | 1 | 13 | 0.171 |
| 22 | 0.761 | 4.092 | 1 | 13 | 0.064 |
| 23 | 0.949 | 0.704 | 1 | 13 | 0.417 |
| 24 | 0.959 | 0.556 | 1 | 13 | 0.469 |
| **Chemical components from urine** |  |  |  |  |  |
| a | 0.861 | 2.098 | 1 | 13 | 0.171 |
| b | 0.903 | 1.401 | 1 | 13 | 0.258 |
| c | 0.948 | 0.716 | 1 | 13 | 0.413 |
| d | 0.898 | 1.473 | 1 | 13 | 0.246 |
| e | 0.999 | 0.008 | 1 | 13 | 0.93 |
| f | 0.969 | 0.419 | 1 | 13 | 0.529 |
| j | 0.999 | 0.019 | 1 | 13 | 0.893 |
| h | 0.94 | 0.832 | 1 | 13 | 0.378 |

**Table A2. Tests of equality of group means (cC57 and BALB).**

|  | **Wilks' Lambda** | **F** | **df1** | **df2** | **Sig.** |
| --- | --- | --- | --- | --- | --- |
| **Chemical components from PGS** |  |  |  |  |  |
| 1 | 0.957 | 0.935 | 1 | 21 | 0.345 |
| 2 | 0.958 | 0.92 | 1 | 21 | 0.348 |
| 3 | 0.783 | 5.812 | 1 | 21 | **0.025** |
| 4 | 0.861 | 3.401 | 1 | 21 | 0.079 |
| 5 | 0.67 | 10.328 | 1 | 21 | **0.004** |
| 6 | 0.88 | 2.865 | 1 | 21 | 0.105 |
| 7 | 0.783 | 5.832 | 1 | 21 | **0.025** |
| 8 | 0.809 | 4.945 | 1 | 21 | **0.037** |
| 9 | 0.986 | 0.289 | 1 | 21 | 0.597 |
| 10 | 0.967 | 0.728 | 1 | 21 | 0.403 |
| 11 | 0.941 | 1.325 | 1 | 21 | 0.263 |
| 12 | 0.901 | 2.313 | 1 | 21 | 0.143 |
| 13 | 0.996 | 0.08 | 1 | 21 | 0.779 |
| 14 | 0.935 | 1.449 | 1 | 21 | 0.242 |
| 15 | 0.873 | 3.06 | 1 | 21 | 0.095 |
| 16 | 0.728 | 7.846 | 1 | 21 | **0.011** |
| 17 | 0.998 | 0.044 | 1 | 21 | 0.836 |
| 18 | 0.093 | 204.581 | 1 | 21 | **0** |
| 19 | 0.2 | 83.831 | 1 | 21 | **0** |
| 20 | 0.901 | 2.32 | 1 | 21 | 0.143 |
| 21 | 0.931 | 1.555 | 1 | 21 | 0.226 |
| 22 | 0.675 | 10.091 | 1 | 21 | **0.005** |
| 23 | 0.639 | 11.853 | 1 | 21 | **0.002** |
| 24 | 0.867 | 3.231 | 1 | 21 | 0.087 |
| **Chemical components from urine** |  |  |  |  |  |
| a | 0.797 | 5.351 | 1 | 21 | **0.031** |
| b | 0.918 | 1.874 | 1 | 21 | 0.185 |
| c | 0.78 | 5.927 | 1 | 21 | **0.024** |
| d | 0.947 | 1.174 | 1 | 21 | 0.291 |
| e | 0.859 | 3.441 | 1 | 21 | 0.078 |
| f | 0.9 | 2.337 | 1 | 21 | 0.141 |
| g | 0.577 | 15.395 | 1 | 21 | **0.001** |
| h | 0.935 | 1.47 | 1 | 21 | 0.239 |

Figures in red are significant.

**Table A3. Classification results of cC57 and BLAB groups based on the significant indexes in table A2.^b,c^**

|  | | **Group** | **Predicted group membership** | | **Total** |
| --- | --- | --- | --- | --- | --- |
|  |  |  | **1** | **2** |  |
| Original | Count | cC57 | 15 | 0 | 15 |
|  |  | BLAB | 0 | 8 | 8 |
|  | % | cC57 | 100 | 0 | 100 |
|  |  | BLAB | 0 | 100 | 100 |
| Cross-validated^a^ | Count | cC57 | 15 | 0 | 15 |
|  |  | BLAB | 0 | 8 | 8 |
|  | % | cC57 | 100 | 0 | 100 |
|  |  | BLAB | 0 | 100 | 100 |

^a^Cross validation is done only for those cases in the analysis. In cross validation, each case is classified by the functions derived from all cases other than that case.

^b^100.0% of original grouped cases correctly classified.

^c^100.0% of cross-validated grouped cases correctly classified.

**Table A4. Discriminant analysis between BLAB and C57 groups based on the significant indexes in table A2.**

| **Tests of equality of group means** | | | | | |
| --- | --- | --- | --- | --- | --- |
|  | **Wilks' Lambda** | **F** | **df1** | **df2** | **Sig.** |
| **Chemical components from PGS** | | |  |  |  |
| 3 | 0.702 | 5.934 | 1 | 14 | **0.029** |
| 5 | 0.49 | 14.599 | 1 | 14 | **0.002** |
| 7 | 0.86 | 2.273 | 1 | 14 | 0.154 |
| 8 | 0.857 | 2.331 | 1 | 14 | 0.149 |
| 16 | 0.738 | 4.965 | 1 | 14 | **0.043** |
| 18 | 0.077 | 168.127 | 1 | 14 | **0** |
| 19 | 0.197 | 57.217 | 1 | 14 | **0** |
| 22 | 0.832 | 2.826 | 1 | 14 | 0.115 |
| 23 | 0.562 | 10.92 | 1 | 14 | **0.005** |
| **Chemical components from urine** | | |  |  |  |
| a | 0.6 | 9.336 | 1 | 14 | **0.009** |
| c | 0.681 | 6.552 | 1 | 14 | **0.023** |
| j | 0.688 | 6.344 | 1 | 14 | **0.025** |

Figures in red are significant.

| **Classification results^b,c^** | | | | | |
| --- | --- | --- | --- | --- | --- |
|  |  | Groups | Predicted Group Membership | | Total |
|  |  |  | 1.00 | 2.00 |  |
| Original | Count | BLAB | 8 | 0 | 8 |
|  |  | C57 | 0 | 8 | 8 |
|  | % | BLAB | 100.0 | .0 | 100.0 |
|  |  | C57 | .0 | 100.0 | 100.0 |
| Cross-validated^a^ | Count | BLAB | 7 | 1 | 8 |
|  |  | C57 | 2 | 6 | 8 |
|  | % | BLAB | 87.5 | 12.5 | 100.0 |
|  |  | C57 | 25.0 | 75.0 | 100.0 |
| ^a^Cross validation is done only for those cases in the analysis. In cross validation, each case is classified by the functions derived from all cases other than that case. | | | | | |
| ^b^100.0% of original grouped cases correctly classified. | | | | | |
| ^c^81.3% of cross-validated grouped cases correctly classified. | | | | | |

**Table A5. Discriminant analysis between BLAB and adopted C57** **groups based on the significant index in table A2.**

| **Tests of equality of group means** | | |  |  |  |
| --- | --- | --- | --- | --- | --- |
|  | **Wilks' Lambda** | **F** | **df1** | **df2** | **Sig.** |
| **Chemical components from PGS** | | |  |  |  |
| 3 | 0.753 | 4.27 | 1 | 13 | 0.059 |
| 5 | 0.734 | 4.708 | 1 | 13 | **0.049** |
| 7 | 0.656 | 6.827 | 1 | 13 | **0.021** |
| 8 | 0.703 | 5.482 | 1 | 13 | **0.036** |
| 16 | 0.609 | 8.329 | 1 | 13 | **0.013** |
| 18 | 0.096 | 122.259 | 1 | 13 | **0** |
| 19 | 0.182 | 58.406 | 1 | 13 | **0** |
| 22 | 0.242 | 40.672 | 1 | 13 | **0** |
| 23 | 0.678 | 6.181 | 1 | 13 | **0.027** |
| **Chemical components from urine** | | | |  |  |
| a | 0.94 | 0.834 | 1 | 13 | 0.378 |
| c | 0.731 | 4.794 | 1 | 13 | **0.047** |
| g | 0.527 | 11.646 | 1 | 13 | **0.005** |

Figures in red are significant.

| **Classification results^b,c^** | | | | | |
| --- | --- | --- | --- | --- | --- |
|  |  | Groups | Predicted Group Membership | | Total |
|  |  |  | 1.00 | 2.00 |  |
| Original | Count | 1.00 | 7 | 0 | 7 |
|  |  | 2.00 | 0 | 8 | 8 |
|  | % | 1.00 | 100.0 | .0 | 100.0 |
|  |  | 2.00 | .0 | 100.0 | 100.0 |
| Cross-validated^a^ | Count | 1.00 | 7 | 0 | 7 |
|  |  | 2.00 | 0 | 8 | 8 |
|  | % | 1.00 | 100.0 | .0 | 100.0 |
|  |  | 2.00 | .0 | 100.0 | 100.0 |
| ^a^Cross validation is done only for those cases in the analysis. In cross validation, each case is classified by the functions derived from all cases other than that case. | | | | | |
| ^b^100.0% of original grouped cases correctly classified. | | | | | |
| ^c^100.0% of cross-validated grouped cases correctly classified. | | | | | |

**Table A6. Discriminant analysis between cC57and BALB groups based on the indexes selected from the second level screening.**

| **Tests of equality of group means** | | | | | | |
| --- | --- | --- | --- | --- | --- | --- |
|  | **Wilks' Lambda** | | **F** | **df1** | **df2** | **Sig.** |
| **Chemical components from PGS** | | | | |  |  |
| 5 | 0.67 | | 10.328 | 1 | 21 | **0.004** |
| 16 | 0.728 | | 7.846 | 1 | 21 | **0.011** |
| 18 | 0.093 | | 204.581 | 1 | 21 | **0** |
| 19 | 0.2 | | 83.831 | 1 | 21 | **0** |
| 23 | 0.639 | | 11.853 | 1 | 21 | **0.002** |
| **Chemical components from urine** | | | | |  |  |
| c | 0.78 | 5.927 | | 1 | 21 | **0.024** |
| g | 0.577 | 15.395 | | 1 | 21 | **0.001** |

Figures in red are significant.

| **Classification results^b,c^** | | | | | |
| --- | --- | --- | --- | --- | --- |
|  |  | Groups | Predicted Group Membership | | Total |
|  |  |  | cC57 | BLAB |  |
| Original | Count | cC57 | 15 | 0 | 15 |
|  |  | BLAB | 0 | 8 | 8 |
|  | % | cC57 | 100.0 | .0 | 100.0 |
|  |  | BLAB | .0 | 100.0 | 100.0 |
| Cross-validated^a^ | Count | cC57 | 15 | 0 | 15 |
|  |  | BLAB | 0 | 8 | 8 |
|  | % | cC57 | 100.0 | .0 | 100.0 |
|  |  | BLAB | .0 | 100.0 | 100.0 |
| ^a^Cross validation is done only for those cases in the analysis. In cross validation, each case is classified by the functions derived from all cases other than that case. | | | | | |
| ^b^100.0% of original grouped cases correctly classified. | | | | | |
| ^c^100.0% of cross-validated grouped cases correctly classified. | | | | | |

**Table A7. Discriminant analysis between BLAB and C57 groups based on the indexes selected from the second level screening.**

| **Tests of equality of group means** | | | | | |
| --- | --- | --- | --- | --- | --- |
|  | **Wilks' Lambda** | **F** | **df1** | **df2** | **Sig.** |
| **Chemical components from PGS** | | | | | |
| 5 | 0.49 | 14.599 | 1 | 14 | **0.002** |
| 16 | 0.738 | 4.965 | 1 | 14 | **0.043** |
| 18 | 0.077 | 168.127 | 1 | 14 | **0** |
| 19 | 0.197 | 57.217 | 1 | 14 | **0** |
| 23 | 0.562 | 10.92 | 1 | 14 | **0.005** |
| **Chemical components from urine** | | | |  |  |
| c | 0.681 | 6.552 | 1 | 14 | **0.023** |
| g | 0.688 | 6.344 | 1 | 14 | **0.025** |

Figures in red are significant.

| **Classification results^b,c^** | | | | | |
| --- | --- | --- | --- | --- | --- |
|  |  | Groups | Predicted Group Membership | | Total |
|  |  |  | 1.00 | 2.00 |  |
| Original | Count | BLAB | 8 | 0 | 8 |
|  |  | C57 | 0 | 8 | 8 |
|  | % | BLAB | 100.0 | .0 | 100.0 |
|  |  | C57 | .0 | 100.0 | 100.0 |
| Cross-validated^a^ | Count | BLAB | 8 | 0 | 8 |
|  |  | C57 | 0 | 8 | 8 |
|  | % | BLAB | 100.0 | .0 | 100.0 |
|  |  | C57 | .0 | 100.0 | 100.0 |
| ^a^Cross validation is done only for those cases in the analysis. In cross validation, each case is classified by the functions derived from all cases other than that case. | | | | | |
| ^b^100.0% of original grouped cases correctly classified. | | | | | |
| ^c^100.0% of cross-validated grouped cases correctly classified. | | | | | |

**Table A8. Discriminant analysis between BLAB and adopted C57 groups based on the indexes selected from the second level screening.**

| **Tests of equality of group means** | | | | | |
| --- | --- | --- | --- | --- | --- |
|  | **Wilks' Lambda** | **F** | **df1** | **df2** | **Sig.** |
| **Chemical components from PGS** | | | | | |
| 5 | 0.734 | 4.708 | 1 | 13 | 0.049 |
| 16 | 0.609 | 8.329 | 1 | 13 | 0.013 |
| 18 | 0.096 | 122.259 | 1 | 13 | 0 |
| 19 | 0.182 | 58.406 | 1 | 13 | 0 |
| 23 | 0.678 | 6.181 | 1 | 13 | 0.027 |
| **Chemical components from urine** | | | | | |
| c | 0.731 | 4.794 | 1 | 13 | 0.047 |
| g | 0.527 | 11.646 | 1 | 13 | 0.005 |

Figures in red are significant.

| **Classification results^b,c^** | | | | | |
| --- | --- | --- | --- | --- | --- |
|  |  | Groups | Predicted Group Membership | | Total |
|  |  |  | 1.00 | 2.00 |  |
| Original | Count | BLAB | 7 | 0 | 7 |
|  |  | Adopted C57 | 0 | 8 | 8 |
|  | % | BLAB | 100.0 | .0 | 100.0 |
|  |  | Adopted C57 | .0 | 100.0 | 100.0 |
| Cross-validated^a^ | Count | BLAB | 7 | 0 | 7 |
|  |  | Adopted C57 | 0 | 8 | 8 |
|  | % | BLAB | 100.0 | .0 | 100.0 |
|  |  | Adopted C57 | .0 | 100.0 | 100.0 |
| ^a^Cross validation is done only for those cases in the analysis. In cross validation, each case is classified by the functions derived from all cases other than that case. | | | | | |
| ^b^100.0% of original grouped cases correctly classified. | | | | | |
| ^c^100.0% of cross-validated grouped cases correctly classified. | | | | | |

**Table A9. Tests of equality of group means (BLAB vs. adopted BLAB).**

|  | **Wilks' Lambda** | **F** | **df1** | **df2** | **Sig.** |
| --- | --- | --- | --- | --- | --- |
| **Chemical components from PGS** | | | | | |
| 1 | 0.85 | 2.48 | 1 | 14 | 0.138 |
| 2 | 0.864 | 2.195 | 1 | 14 | 0.161 |
| 3 | 0.993 | 0.094 | 1 | 14 | 0.764 |
| 4 | 0.985 | 0.214 | 1 | 14 | 0.651 |
| 5 | 0.955 | 0.653 | 1 | 14 | 0.432 |
| 6 | 0.979 | 0.302 | 1 | 14 | 0.591 |
| 7 | 0.985 | 0.213 | 1 | 14 | 0.652 |
| 8 | 0.961 | 0.563 | 1 | 14 | 0.466 |
| 9 | 0.972 | 0.4 | 1 | 14 | 0.537 |
| 10 | 0.977 | 0.331 | 1 | 14 | 0.574 |
| 11 | 0.7 | 5.996 | 1 | 14 | **0.028** |
| 12 | 1 | 0.006 | 1 | 14 | 0.938 |
| 13 | 0.946 | 0.795 | 1 | 14 | 0.388 |
| 14 | 0.995 | 0.066 | 1 | 14 | 0.801 |
| 15 | 0.962 | 0.56 | 1 | 14 | 0.467 |
| 16 | 0.933 | 1.01 | 1 | 14 | 0.332 |
| 17 | 0.951 | 0.717 | 1 | 14 | 0.411 |
| 18 | 0.902 | 1.521 | 1 | 14 | 0.238 |
| 19 | 0.964 | 0.515 | 1 | 14 | 0.485 |
| 20 | 0.999 | 0.019 | 1 | 14 | 0.894 |
| 21 | 0.989 | 0.158 | 1 | 14 | 0.697 |
| 22 | 0.944 | 0.834 | 1 | 14 | 0.376 |
| 23 | 0.907 | 1.44 | 1 | 14 | 0.25 |
| 24 | 0.93 | 1.055 | 1 | 14 | 0.322 |
| **Chemical components from urine** | | | | | |
| a | 0.969 | 0.45 | 1 | 14 | 0.513 |
| b | 0.945 | 0.816 | 1 | 14 | 0.382 |
| c | 0.924 | 1.156 | 1 | 14 | 0.301 |
| d | 0.965 | 0.507 | 1 | 14 | 0.488 |
| e | 0.89 | 1.726 | 1 | 14 | 0.21 |
| f | 0.995 | 0.07 | 1 | 14 | 0.795 |
| g | 0.998 | 0.022 | 1 | 14 | 0.884 |
| h | 0.942 | 0.862 | 1 | 14 | 0.369 |

**Table A10. Tests of equality of group means (cBALB vs. C57).**

|  | **Wilks' Lambda** | **F** | **df1** | **df2** | **Sig.** |
| --- | --- | --- | --- | --- | --- |
| **Chemical components from PGS** | | | |  |  |
| 1 | 0.935 | 1.539 | 1 | 22 | 0.228 |
| 2 | 0.937 | 1.476 | 1 | 22 | 0.237 |
| 3 | 0.707 | 9.099 | 1 | 22 | **0.006** |
| 4 | 0.795 | 5.689 | 1 | 22 | **0.026** |
| 5 | 0.512 | 20.956 | 1 | 22 | **0** |
| 6 | 0.856 | 3.71 | 1 | 22 | 0.067 |
| 7 | 0.839 | 4.234 | 1 | 22 | 0.052 |
| 8 | 0.825 | 4.682 | 1 | 22 | **0.042** |
| 9 | 0.98 | 0.449 | 1 | 22 | 0.51 |
| 10 | 0.958 | 0.96 | 1 | 22 | 0.338 |
| 11 | 0.94 | 1.4 | 1 | 22 | 0.249 |
| 12 | 0.804 | 5.349 | 1 | 22 | **0.03** |
| 13 | 0.995 | 0.113 | 1 | 22 | 0.74 |
| 14 | 0.782 | 6.12 | 1 | 22 | **0.022** |
| 15 | 0.818 | 4.901 | 1 | 22 | **0.038** |
| 16 | 0.801 | 5.48 | 1 | 22 | **0.029** |
| 17 | 0.998 | 0.048 | 1 | 22 | 0.828 |
| 18 | 0.131 | 145.393 | 1 | 22 | **0** |
| 19 | 0.232 | 72.692 | 1 | 22 | **0** |
| 20 | 0.88 | 3.005 | 1 | 22 | 0.097 |
| 21 | 0.809 | 5.205 | 1 | 22 | **0.033** |
| 22 | 0.763 | 6.828 | 1 | 22 | **0.016** |
| 23 | 0.638 | 12.492 | 1 | 22 | **0.002** |
| 24 | 0.842 | 4.128 | 1 | 22 | 0.054 |
| **Chemical components from urine** | | | |  |  |
| a | 0.63 | 12.925 | 1 | 22 | **0.002** |
| b | 0.826 | 4.635 | 1 | 22 | **0.043** |
| c | 0.669 | 10.908 | 1 | 22 | **0.003** |
| d | 0.807 | 5.277 | 1 | 22 | **0.032** |
| e | 0.888 | 2.776 | 1 | 22 | 0.11 |
| f | 0.912 | 2.11 | 1 | 22 | 0.16 |
| g | 0.74 | 7.746 | 1 | 22 | **0.011** |
| h | 0.985 | 0.335 | 1 | 22 | 0.568 |

Figures in red are significant.

**Table A11. Discriminant analysis between cBALB and C57 groups based on the significant index in table A10.**

| **Tests of equality of group means** | | | | | | |
| --- | --- | --- | --- | --- | --- | --- |
|  | **Wilks' Lambda** | | **F** | **df1** | **df2** | **Sig.** |
| **Chemical components from PGS** | | | |  |  |  |
| 3 | 0.707 | 9.099 | | 1 | 22 | **0.006** |
| 4 | 0.795 | 5.689 | | 1 | 22 | **0.026** |
| 5 | 0.512 | 20.956 | | 1 | 22 | **0** |
| 8 | 0.825 | 4.682 | | 1 | 22 | **0.042** |
| 12 | 0.804 | 5.349 | | 1 | 22 | **0.03** |
| 14 | 0.782 | 6.12 | | 1 | 22 | **0.022** |
| 15 | 0.818 | 4.901 | | 1 | 22 | **0.038** |
| 16 | 0.801 | 5.48 | | 1 | 22 | **0.029** |
| 18 | 0.131 | 145.393 | | 1 | 22 | **0** |
| 19 | 0.232 | 72.692 | | 1 | 22 | **0** |
| 21 | 0.809 | 5.205 | | 1 | 22 | **0.033** |
| 22 | 0.763 | 6.828 | | 1 | 22 | **0.016** |
| 23 | 0.638 | 12.492 | | 1 | 22 | **0.002** |
| **Chemical components from urine** | | | |  |  |  |
| a | 0.63 | 12.925 | | 1 | 22 | **0.002** |
| b | 0.826 | 4.635 | | 1 | 22 | **0.043** |
| c | 0.669 | 10.908 | | 1 | 22 | **0.003** |
| d | 0.807 | 5.277 | | 1 | 22 | **0.032** |
| g | 0.74 | 7.746 | | 1 | 22 | **0.011** |

Figures in red are significant.

| **Classification results^b,c^** | | | | | |
| --- | --- | --- | --- | --- | --- |
|  |  | Groups | Predicted Group Membership | | Total |
|  |  |  | 1.00 | 2.00 |  |
| Original | Count | 1.00 | 16 | 0 | 16 |
|  |  | 2.00 | 0 | 8 | 8 |
|  | % | 1.00 | 100.0 | .0 | 100.0 |
|  |  | 2.00 | .0 | 100.0 | 100.0 |
| Cross-validated^a^ | Count | 1.00 | 16 | 0 | 16 |
|  |  | 2.00 | 1 | 7 | 8 |
|  | % | 1.00 | 100.0 | .0 | 100.0 |
|  |  | 2.00 | 12.5 | 87.5 | 100.0 |
| ^a^Cross validation is done only for those cases in the analysis. In cross validation, each case is classified by the functions derived from all cases other than that case. | | | | | |
| ^b^100.0% of original grouped cases correctly classified. | | | | | |
| ^c^95.8% of cross-validated grouped cases correctly classified. | | | | | |

**Table A12. Discriminant analysis between C57 and BALB groups based on the significant index in table A10.**

| **Tests of equality of group means** | | | | | |
| --- | --- | --- | --- | --- | --- |
|  | **Wilks' Lambda** | **F** | **df1** | **df2** | **Sig.** |
| **Chemical components from PGS** | | | | | |
| 3 | 0.702 | 5.934 | 1 | 14 | **0.029** |
| 4 | 0.784 | 3.857 | 1 | 14 | **0.07** |
| 5 | 0.49 | 14.599 | 1 | 14 | **0.002** |
| 8 | 0.857 | 2.331 | 1 | 14 | 0.149 |
| 12 | 0.829 | 2.895 | 1 | 14 | 0.111 |
| 14 | 0.829 | 2.884 | 1 | 14 | 0.112 |
| 15 | 0.871 | 2.077 | 1 | 14 | 0.172 |
| 16 | 0.738 | 4.965 | 1 | 14 | **0.043** |
| 18 | 0.077 | 168.127 | 1 | 14 | **0** |
| 19 | 0.197 | 57.217 | 1 | 14 | **0** |
| 21 | 0.852 | 2.437 | 1 | 14 | 0.141 |
| 22 | 0.832 | 2.826 | 1 | 14 | 0.115 |
| 23 | 0.562 | 10.92 | 1 | 14 | **0.005** |
| **Chemical components from urine** | | | | | |
| a | 0.6 | 9.336 | 1 | 14 | **0.009** |
| b | 0.853 | 2.404 | 1 | 14 | 0.143 |
| c | 0.681 | 6.552 | 1 | 14 | **0.023** |
| d | 0.813 | 3.223 | 1 | 14 | 0.094 |
| g | 0.688 | 6.344 | 1 | 14 | **0.025** |

Figures in red are significant.

| **Classification results^b,c^** | | | | | |
| --- | --- | --- | --- | --- | --- |
|  |  | Groups | Predicted Group Membership | | Total |
|  |  |  | 1.00 | 2.00 |  |
| Original | Count | C57 | 8 | 0 | 8 |
|  |  | BALB | 0 | 8 | 8 |
|  | % | C57 | 100.0 | .0 | 100.0 |
|  |  | BALB | .0 | 100.0 | 100.0 |
| Cross-validated^a^ | Count | C57 | 7 | 1 | 8 |
|  |  | BALB | 2 | 6 | 8 |
|  | % | C57 | 87.5 | 12.5 | 100.0 |
|  |  | BALB | 25.0 | 75.0 | 100.0 |
| ^a^Cross validation is done only for those cases in the analysis. In cross validation, each case is classified by the functions derived from all cases other than that case. | | | | | |
| ^b^100.0% of original grouped cases correctly classified. | | | | | |
| ^c^81.3% of cross-validated grouped cases correctly classified. | | | | | |

**Table A13. Discriminant analysis between C57 and adopted BALB groups based on the significant index in table A10.**

| **Tests of equality of group means** | | | | | |
| --- | --- | --- | --- | --- | --- |
|  | **Wilks' Lambda** | **F** | **df1** | **df2** | **Sig.** |
| **Chemical components from PGS** | | | | | |
| 3 | 0.751 | 4.638 | 1 | 14 | **0.049** |
| 4 | 0.838 | 2.706 | 1 | 14 | 0.122 |
| 5 | 0.554 | 11.274 | 1 | 14 | **0.005** |
| 8 | 0.742 | 4.862 | 1 | 14 | **0.045** |
| 12 | 0.824 | 2.989 | 1 | 14 | 0.106 |
| 14 | 0.821 | 3.06 | 1 | 14 | 0.102 |
| 15 | 0.827 | 2.931 | 1 | 14 | 0.109 |
| 16 | 0.862 | 2.249 | 1 | 14 | 0.156 |
| 18 | 0.11 | 112.926 | 1 | 14 | **0** |
| 19 | 0.216 | 50.832 | 1 | 14 | **0** |
| 21 | 0.846 | 2.548 | 1 | 14 | 0.133 |
| 22 | 0.719 | 5.467 | 1 | 14 | **0.035** |
| 23 | 0.671 | 6.866 | 1 | 14 | **0.02** |
| **Chemical components from urine** | | | | | |
| a | 0.486 | 14.783 | 1 | 14 | **0.002** |
| b | 0.872 | 2.064 | 1 | 14 | 0.173 |
| c | 0.751 | 4.631 | 1 | 14 | **0.049** |
| d | 0.672 | 6.824 | 1 | 14 | **0.02** |
| g | 0.66 | 7.22 | 1 | 14 | **0.018** |

Figures in red are significant.

**Classification results^b,c^**

|  |  | Groups | Predicted Group Membership | | Total |
| --- | --- | --- | --- | --- | --- |
|  |  |  | 1.00 | 2.00 |  |
| Original | Count | C57 | 8 | 0 | 8 |
|  |  | Adopted BALB | 0 | 8 | 8 |
|  | % | C57 | 100.0 | .0 | 100.0 |
|  |  | Adopted BALB | .0 | 100.0 | 100.0 |
| Cross-validated^a^ | Count | C57 | 7 | 1 | 8 |
|  |  | Adopted BALB | 3 | 5 | 8 |
|  | % | C57 | 87.5 | 12.5 | 100.0 |
|  |  | Adopted BALB | 37.5 | 62.5 | 100.0 |
| ^a^Cross validation is done only for those cases in the analysis. In cross validation, each case is classified by the functions derived from all cases other than that case. | | | | | |
| ^b^100.0% of original grouped cases correctly classified. | | | | | |
| ^c^75.0% of cross-validated grouped cases correctly classified. | | | | | |

**Table A14. Discriminant analysis between cBLAB and C57 groups based on the indexes selected from the second level screening.**

| **Tests of equality of group means** | | | | | |
| --- | --- | --- | --- | --- | --- |
|  | **Wilks' Lambda** | **F** | **df1** | **df2** | **Sig.** |
| **Chemical components from PGS** | | | |  |  |
| 3 | 0.707 | 9.099 | 1 | 22 | **0.006** |
| 5 | 0.512 | 20.956 | 1 | 22 | **0** |
| 18 | 0.131 | 145.393 | 1 | 22 | **0** |
| 19 | 0.232 | 72.692 | 1 | 22 | **0** |
| 23 | 0.638 | 12.492 | 1 | 22 | **0.002** |
| **Chemical components from urine** | | | | | |
| a | 0.63 | 12.925 | 1 | 22 | **0.002** |
| c | 0.669 | 10.908 | 1 | 22 | **0.003** |
| g | 0.74 | 7.746 | 1 | 22 | **0.011** |

Figures in red are significant.

| **Classification results^b,c^** | | | | | |
| --- | --- | --- | --- | --- | --- |
|  |  | Groups | Predicted Group Membership | | Total |
|  |  |  | 1.00 | 2.00 |  |
| Original | Count | cBLAB | 16 | 0 | 16 |
|  |  | C57 | 0 | 8 | 8 |
|  | % | cBLAB | 100.0 | .0 | 100.0 |
|  |  | C57 | .0 | 100.0 | 100.0 |
| Cross-validated^a^ | Count | cBLAB | 16 | 0 | 16 |
|  |  | C57 | 0 | 8 | 8 |
|  | % | cBLAB | 100.0 | .0 | 100.0 |
|  |  | C57 | .0 | 100.0 | 100.0 |
| ^a^Cross validation is done only for those cases in the analysis. In cross validation, each case is classified by the functions derived from all cases other than that case. | | | | | |
| ^b^100.0% of original grouped cases correctly classified. | | | | | |
| ^c^100.0% of cross-validated grouped cases correctly classified. | | | | | |

**Table A15. Discriminant analysis between BLAB and C57 groups based on the indexes selected from the second level screening.**

**Tests of equality of group means.**

|  | **Wilks' Lambda** | **F** | **df1** | **df2** | **Sig.** |
| --- | --- | --- | --- | --- | --- |
| **Chemical components from PGS** | | | |  |  |
| 3 | 0.702 | 5.934 | 1 | 14 | **0.029** |
| 5 | 0.49 | 14.599 | 1 | 14 | **0.002** |
| 18 | 0.077 | 168.127 | 1 | 14 | **0** |
| 19 | 0.197 | 57.217 | 1 | 14 | **0** |
| 23 | 0.562 | 10.92 | 1 | 14 | **0.005** |
| **Chemical components from urine** | | | |  |  |
| a | 0.6 | 9.336 | 1 | 14 | **0.009** |
| c | 0.681 | 6.552 | 1 | 14 | **0.023** |
| g | 0.688 | 6.344 | 1 | 14 | **0.025** |

Figures in red are significant.

| **Classification results^b,c^** | | | | | |
| --- | --- | --- | --- | --- | --- |
|  |  | Groups | Predicted Group Membership | | Total |
|  |  |  | 1.00 | 2.00 |  |
| Original | Count | BLAB | 8 | 0 | 8 |
|  |  | C57 | 0 | 8 | 8 |
|  | % | BLAB | 100.0 | .0 | 100.0 |
|  |  | C57 | .0 | 100.0 | 100.0 |
| Cross-validated^a^ | Count | BLAB | 8 | 0 | 8 |
|  |  | C57 | 0 | 8 | 8 |
|  | % | BLAB | 100.0 | .0 | 100.0 |
|  |  | C57 | .0 | 100.0 | 100.0 |
| ^a^Cross validation is done only for those cases in the analysis. In cross validation, each case is classified by the functions derived from all cases other than that case. | | | | | |
| ^b^100.0% of original grouped cases correctly classified. | | | | | |
| ^c^100.0% of cross-validated grouped cases correctly classified. | | | | | |

**Table A16. Discriminant analysis between BALB and adopted BALB groups based on the indexes selected from the second level screening.**

| **Tests of equality of group means** | | | | | |
| --- | --- | --- | --- | --- | --- |
|  | **Wilks' Lambda** | **F** | **df1** | **df2** | **Sig.** |
| **Chemical components from PGS** | | |  |  |  |
| 3 | 0.751 | 4.638 | 1 | 14 | **0.049** |
| 5 | 0.554 | 11.274 | 1 | 14 | **0.005** |
| 18 | 0.11 | 112.926 | 1 | 14 | **0** |
| 19 | 0.216 | 50.832 | 1 | 14 | **0** |
| 23 | 0.671 | 6.866 | 1 | 14 | **0.02** |
| **Chemical components from urine** | | | |  |  |
| a | 0.486 | 14.783 | 1 | 14 | **0.002** |
| c | 0.751 | 4.631 | 1 | 14 | **0.049** |
| g | 0.66 | 7.22 | 1 | 14 | **0.018** |

Figures in red are significant.

| **Classification results^b,c^** | | | | | |
| --- | --- | --- | --- | --- | --- |
|  |  | Groups | Predicted Group Membership | | Total |
|  |  |  | 1.00 | 2.00 |  |
| Original | Count | BALB | 8 | 0 | 8 |
|  |  | Adopted BALB | 0 | 8 | 8 |
|  | % | BALB | 100.0 | .0 | 100.0 |
|  |  | Adopted BALB | .0 | 100.0 | 100.0 |
| Cross-validated^a^ | Count | BALB | 7 | 1 | 8 |
|  |  | Adopted BALB | 0 | 8 | 8 |
|  | % | BALB | 87.5 | 12.5 | 100.0 |
|  |  | Adopted BALB | .0 | 100.0 | 100.0 |
| ^a^Cross validation is done only for those cases in the analysis. In cross validation, each case is classified by the functions derived from all cases other than that case. | | | | | |
| ^b^100.0% of original grouped cases correctly classified. | | | | | |
| ^c^93.8% of cross-validated grouped cases correctly classified. | | | | | |
