# Supplementary material for: Cross-Fostering of Male Mice Subtly Affects Female Olfactory Preferences
Source: PLoS One. 2016 Jan 12;11(1):e0146662. doi: 10.1371/journal.pone.0146662 (PMC4710493; doi:10.1371/journal.pone.0146662)
Supplement: S1 Table — (DOC) [file pone.0146662.s003.doc]

**Table S1.** Relative abundances of PGSs and urinary volatiles in the four groups of male mice.

| ***No.** | **Compounds** | **BALB (n=8)** | | **C57** | |
| --- | --- | --- | --- | --- | --- |
|  |  | **Control** | **Adopted** | **Control (n=8)** | **Adopted (n=7)** |
| **PGS** |  |  |  |  |  |
| 1 | E-β-farnesene | 6.00±1.50 | 7.19±1.53 | 5.72±1.68 | 5.04±0.80 |
| 2 | E,E-α-farnesene | 3.26±0.93 | 3.96±0.96 | 3.08±1.02 | 2.68±0.54 |
| 3 | Z-7-tetradecen-1-ol | 1.00±0.51 | 1.08±0.61 | 2.05±1.11 | 1.63±0.67 |
| 4 | 1-Tetradecanol | 0.43±0.21 | 0.48±0.24 | 0.79±0.46 | 0.62±0.29 |
| 5 | Z-5-tetradecenol acetate | 0.51±0.12 | 0.45±0.14 | 0.81±0.22 | 0.66±0.23 |
| 6 | 1-Tetradecanol, acetate | 0.61±0.16 | 0.65±0.11 | 0.74±0.12 | 0.69±0.15 |
| 7 | 11-Hexadecen-1-ol, (Z)- | 0.17±0.07 | 0.18±0.06 | 0.12±0.06 | 0.08±0.05 |
| 8 | Branched 1-hexadecanol | 0.46±0.10 | 0.50±0.11 | 0.38±0.10 | 0.33±0.11 |
| 9 | 9-Hexadecen-1-ol, (Z)- | 1.70±0.37 | 1.82±0.41 | 1.65±0.36 | 1.56±0.44 |
| 10 | 1-Hexadecanol | 19.5±2.97 | 20.4±3.04 | 18.7±2.97 | 17.9±3.81 |
| 11 | 1-Pentadecanol acetate | 0.25±0.02 | 0.27±0.02 | 0.25±0.04 | 0.22±0.03 |
| 12 | Z-9-hexadecen-1-ol acetate | 1.12±0.29 | 1.11±0.31 | 1.58±0.70 | 1.29±0.31 |
| 13 | 1-Heptadecanol (branched ?) | 0.73±0.04 | 0.75±0.06 | 0.84±1.23 | 0.40±0.08 |
| 14 | 1-Hexadecanol, acetate (branched) | 1.30±0.39 | 1.26±0.34 | 2.99±2.78 | 1.39±0.60 |
| 15 | Isomer of 11-hexadecen-1-ol, acetate, (Z)- | 4.92±0.39 | 4.79±0.32 | 5.68±1.44 | 5.61±0.75 |
| 16 | 11-Hexadecen-1-ol, acetate, (Z)- | 2.40±0.20 | 2.31±0.18 | 2.13±0.29 | 2.16±0.11 |
| 17 | 1-Hexadecanol, acetate | 45.8±5.15 | 43.6±5.32 | 44.1±8.52 | 49.2±6.27 |
| 18 | 1-Heptadecanol, acetate | 0.25±0.02 | 0.27±0.04 | 0.11±0.02 | 0.12±0.02 |
| 19 | 1-Heptadecanol, acetate (branched)? | 3.43±0.40 | 3.29±0.37 | 1.97±0.37 | 2.18±0.17 |
| 20 | 1-Octadecanol | 0.67±0.17 | 0.66±0.14 | 0.54±0.17 | 0.57±0.16 |
| 21 | 1-Heptadecanol, acetate | 0.13±0.16 | 0.11±0.10 | 1.42±2.33 | 0.34±0.28 |
| 22 | Dodecyl octanoate | 1.06±0.18 | 0.98±0.16 | 1.29±0.33 | 1.71±0.21 |
| 23 | Z-7-octadecen-1-ol acetate | 1.39±0.27 | 1.59±0.38 | 0.95±0.39 | 1.17±0.26 |
| 24 | Octadecyl acetate | 2.75±0.63 | 2.47±0.46 | 2.13±0.54 | 2.49±0.53 |
| **Urine** |  |  |  |  |  |
| a | Z-5,5-dimethyl-2-ethylidenetetrahydrofuran | 6.66±2.25 | 7.43±2.32 | 4.07±0.85 | 5.39±1.84 |
| b | 5-Hepten-2-one | 0.09±0.07 | 0.13±0.10 | 0.64±1.00 | 0.30±0.29 |
| c | 2-Heptanone | 0.37±0.17 | 0.49±0.26 | 1.23±0.93 | 1.07±0.76 |
| d | E-5,5-dimethyl-2-ethylidenetetrahydrofuran | 2.85±1.19 | 3.29±1.27 | 2.04±0.45 | 2.66±1.02 |
| e | Dimethyl sulfone | 4.48±2.41 | 6.64±3.98 | 8.17±4.10 | 5.85±2.40 |
| f | 6-Hydroxy-6-methyl-3-heptanone and  5,5-dimethyl-2-ethyltetrahydrofuran-2-ol | 63.00±9.42 | 61.8±8.81 | 68.17±9.72 | 70.2±9.05 |
| g | R,R-3,4-dehydro-exo-brevicomin | 8.02±4.20 | 8.33±4.24 | 3.92±1.91 | 2.58±1.07 |
| h | (s)-2-sec-butyl-4,5-dihydrothiazole | 14.5±6.48 | 11.9±4.72 | 11.76±5.62 | 11.9±4.59 |

*The numbers and letters correspond to the chromatograph peaks, as shown in Fig. 1.
